# The status *Candidatus* for uncultured taxa of *Bacteria* and *Archaea*: SWOT analysis

**DOI:** 10.1099/ijsem.0.005000

**Published:** 2021-09-13

**Authors:** Mark J. Pallen

**Affiliations:** ^1^​ University of East Anglia, Norwich Research Park, Norwich, UK; ^2^​ Quadram Institute Bioscience, Norwich Research Park, Norwich, UK; ^3^​ School of Veterinary Medicine, University of Surrey, Guildford, Surrey, UK

**Keywords:** bacterial nomenclature, *Candidatus*, genome-based taxonomy, uncultured bacteria

## Abstract

The status *Candidatus* was introduced to bacterial taxonomy in the 1990s to accommodate uncultured taxa defined by analyses of DNA sequences. Here I review the strengths, weaknesses, opportunities and threats (SWOT) associated with the status *Candidatus* in the light of a quarter century of use, twinned with recent developments in bacterial taxonomy and sequence-based taxonomic discovery. Despite ambiguities as to its scope, philosophical objections to its use and practical problems in implementation, the status *Candidatus* has now been applied to over 1000 taxa and has been widely adopted by journals and databases. Although lacking priority under the International Code for Nomenclature of Prokaryotes, many *Candidatus* names have already achieved *de facto* standing in the academic literature and in databases via description of a taxon in a peer-reviewed publication, alongside deposition of a genome sequence and there is a clear path to valid publication of such names on culture. Continued and increased use of *Candidatus* names provides an alternative to the potential upheaval that might accompany creation of a new additional code of nomenclature and provides a ready solution to the urgent challenge of naming many thousands of newly discovered but uncultured species.

## Introduction

The International Code for Nomenclature of Prokaryotes (the ICNP or ‘the Code’) articulates principles, rules and recommendations for the naming of archaeal and bacterial taxa, including rules for the valid publication of names and establishing priority in the literature [1]. The current version of the Code is the descendent of earlier documents, stretching back to the 1860s [2, 3]. A major landmark in the development of the Code was the publication of the Approved List of Bacterial Names [4], which brought order out of chaos in the use of names for bacterial taxa, jettisoning over 20000 names, while giving priority to around 2300 names with effect from 1 January 1980 [5]. However, over 50 of these approved names were applied to species that had not, and still have not, been isolated in axenic culture, so that no type strain exists (Table 1), which means that these names could not be validly published if presented for the first time under today’s rules.

**Table 1.** Species in the Approved List of Bacterial Names for which no cultured type strain is available This list was compiled by downloading the genera, species and subspecies list from the List of Prokaryotic names with Standing in Nomenclature (https://lpsn.dsmz.de/downloads) and then sorting and selecting entries by nomenclatural type.

Species	LPSN description of type strain
* Achromatium oxaliferum *	No culture isolated
* Aegyptianella pullorum *	No culture isolated
* Anaplasma marginale *	No culture isolated
* Anaplasma ovis *	No culture isolated
* Ancalochloris perfilievii *	No pure culture
* Bactoderma alba *	No culture available
* Blastobacter henricii *	No culture isolated
* Blattabacterium cuenoti *	No culture isolated
* Borrelia anserina *	No culture available
* Borrelia brasiliensis *	No culture available
* Borrelia caucasica *	No culture available
* Borrelia crocidurae *	No culture available
* Borrelia dugesii *	No culture available
* Borrelia duttonii *	No culture available
* Borrelia graingeri *	No culture available
* Borrelia harveyi *	No culture available
* Borrelia hermsii *	No culture available
* Borrelia hispanica *	No culture available
* Borrelia latyschewii *	No culture available
* Borrelia mazzottii *	No culture available
* Borrelia parkeri *	No culture available
* Borrelia persica *	No culture available
* Borrelia recurrentis *	No culture available
* Borrelia theileri *	No culture available
* Borrelia tillae *	No culture available
* Borrelia turicatae *	No culture available
* Borrelia venezuelensis *	No culture available
* Chloronema giganteum *	No pure culture
* Crenothrix polyspora *	No culture isolated
* Cristispira pectinis *	No culture isolated
* Eperythrozoon coccoides *	No culture isolated
* Eperythrozoon parvum *	No culture isolated
* Gallionella ferruginea *	No culture isolated
* Leptothrix ochracea *	No culture available
* Macromonas mobilis *	No culture available
* Mycobacterium leprae *	Has not been cultivated
* Mycobacterium lepraemurium *	None specified due to difficulties in cultivation
* Neorickettsia helminthoeca *	No culture isolated
* Nitrosococcus nitrosus *	No culture isolated
* Nitrosospira briensis *	No culture available
* Oscillospira guilliermondii *	No culture isolated
* Pasteuria ramosa *	Description from 1888 serves as type
* Planctomyces bekefii *	No culture isolated
* Rickettsiella popilliae *	No culture isolated
* Rickettsiella stethorae *	No culture isolated
* Spirochaeta plicatilis *	No culture available
* Symbiotes lectularius *	No culture isolated
* Thiopedia rosea *	No pure culture
* Thioploca schmidlei *	No culture isolated
* Thiospira winogradskyi *	No culture available
* Thiovulum majus *	No culture isolated
* Toxothrix trichogenes *	No culture isolated
* Treponema pallidum *	No culture available; none designated
* Treponema paraluiscuniculi *	No culture available
* Treponema pertenue *	No culture available; none designated
* Wolbachia melophagi *	No culture isolated
* Wolbachia pipientis *	No culture isolated

In the 1980s and 1990s, there was a growing recognition that many, if not most, bacterial taxa could not be isolated or maintained in axenic culture, but could be identified and classified through analysis of macromolecular sequences [6–8]. To accommodate this new source of taxonomic information, in 1994 Murray and Schleifer proposed a new category of taxonomic name, which they called *Candidatus*, to ‘provide a proper record of sequence-based potential new taxa’ [9].

After consideration by the International Committee on Systematics of Bacteria, additional guidelines were published in 1995 [10]. Curiously, although the committee agreed that a ‘*Candidatus* name is by definition a preliminary name and therefore has no standing in prokaryote nomenclature’, they were happy to incorporate the *Candidatus* option within an appendix within the Code and suggested that a list of organisms with the status *Candidatus* should maintained and published in the *International Journal of Systematic and Evolutionary Microbiology* (IJSEM) at appropriate intervals [1, 11]. In parallel with these developments, the Committee discussed and then mandated a requirement that valid publication of names required deposition of viable pure cultures in strain repositories in two countries, which came into effect from 1 January 2001 [1, 11, 12].

Recent years have seen a dramatic growth in the discovery and classification of new uncultured taxa primarily through analysis of metagenomic sequences [13–15]. This has prompted calls for a change to the Code to give standing to names for uncultured taxa [16, 17]. A proposal to enable this was discussed and rejected by the International Committee on Systematics of Prokaryotes in 2020 [18]. This in turn has fuelled calls for the establishment of an additional or alternative code of nomenclature for uncultured microbes called the SeqCode [17, 19]. However, the *Candidatus* option has already been adopted for uncultured organisms [10, 20]. So, this begs the question: if the system works, do we actually need to change anything? To inform thinking on this issue, here I present a SWOT analysis, evaluating the strengths, weaknesses, opportunities and threats pertaining to the status *Candidatus*.

## Strengths

Key strengths of the status *Candidatus* are that it has been in place for over a quarter of a century and that its use is already specified and permitted within the current version of the Code: ‘This category should be used for describing prokaryotic entities for which more than a mere nucleic acid sequence is available but for which characteristics required for description according to the Code are lacking’. Furthermore, the way in which *Candidatus* taxa are described has evolved over time. Thus, the examples provided in the original guidelines (copied over into Appendix 11 of the Code) failed to comply with the grammatical or orthographic norms of bacterial taxonomic nomenclature (e.g. adjectives and nouns agree in gender; connecting vowels are used consistently, binomials are specified). However, with the occasional exception—such as the orphaned species epithet *Candidatus* comitans [21]—*Candidatus* names have since then followed the conventions of Linnaean nomenclature, for example in specifying binomials for species or using relevant endings for proposed families (-*aceae*) or orders (-*ales*) [20].

**Fig. 1.**
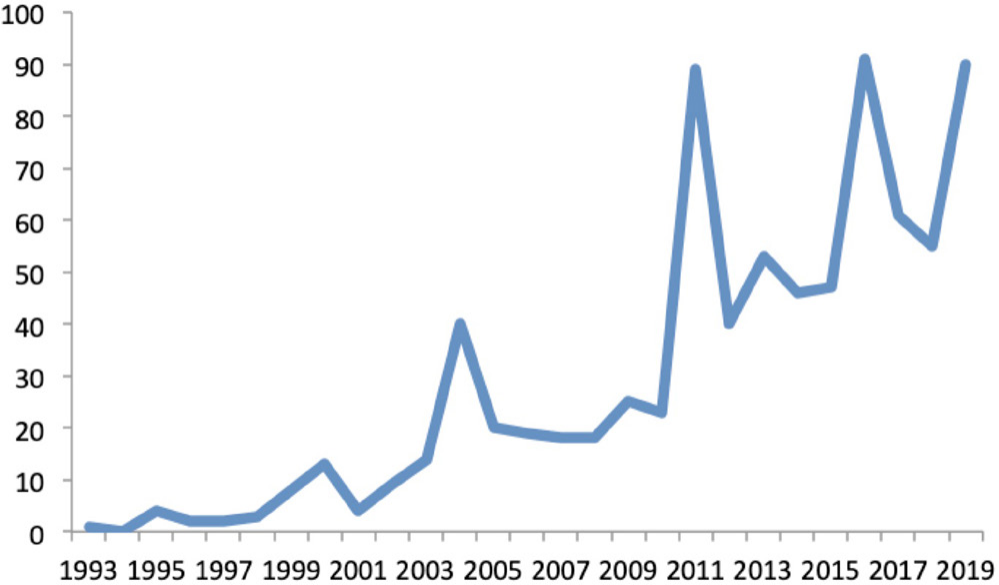
Number of *Candidatus* species names published each calendar year 1993–2019. Data extracted from published *Candidatus* lists [20, 22].

*Candidatus* designations have seen increasing usage over the last 25 years (Fig. 1). Lists of *Candidatus* names published from 1995 to 2019 document over 1000 names in total, including more than 700 species [20, 22]. By contrast, an alternative suggestion of using a superscripted ‘u’ to mark names of uncultured organisms has been adopted only a handful of times [16, 23, 24].

A PubMed search reveals that the term *Candidatus* has been used in over 500 journals, suggesting widespread acceptance among authors, reviewers and editors. In addition, the term *Candidatus* remains unambiguous, so that searches of the web or of the biomedical literature with this term return only appropriate hits. Crucially, *Candidatus* names are included in—and indeed often enforced by—the widely used NCBI taxonomy [25] and are documented in the List of Prokaryotic names with Standing in Nomenclature (LPSN) [26] and by the NamesforLife service [27]. However, it is worth noting the qualifier *Candidatus* is ignored by the widely used Genome Taxonomy Database (GTDB), which even goes so far as to strip the term off names imported from elsewhere [28].

Another potential strength is that the *Candidatus* approach provides a clear route to valid publication of names if taxa are subsequently cultured—in such circumstances, the term *Candidatus* can simply be deleted from the existing name. Over 30 *Candidatus* species have now been cultured and with existing names carried over, minus the term *Candidatus* and with occasional linguistic corrections [20, 22], with just one exception. And in that exceptional case, the re-designation of *Candidatus* Pectobacterium maceratum as *Pectobacterium versatile* was not based on a whim, but instead justified by the authors by reference to Recommendation 12c in the Code, aiming to avoid epithets based on a character common to all species within a genus [29].

## Weaknesses

A key weakness is that the circumstances governing use of *Candidatus* status are poorly specified in the Code—rather than defined by Rules or Recommendations within the main body, the guidance is relegated to an Appendix—and so it remains unclear when use is allowed or denied, mandatory or merely optional. In fact, there has been confusion as to whether the status *Candidatus* can be used for taxa that have been cultured but not adequately characterized or is restricted to uncultured taxa. Thus, the term *Candidatus* has been applied to 20 or more taxa that had already been propagated in pure culture (or at least in culture free of other bacteria) at the time the description was published [20]. Although the original proposals for the use of *Candidatus* [10]—and recent expert opinion [20]—suggest it should apply only to uncultured taxa, Appendix 11 in the Code does not mention culture or lack of culture, but merely states: ‘This category should be used for describing prokaryotic entities … for which characteristics required for description according to the Code are lacking’. However, as we shall see later, this ambiguity might prove useful.

As the Code is also ambiguous on the issue of whether type strains have to be maintained in pure culture, it remains unclear whether the status *Candidatus* can be applied when viable but impure cultures of a bacterium or archaeon are available and could be deposited. Rule 18a states ‘The type strain is made up of living cultures of an organism, which … *should* have been maintained in pure culture’—note *should* rather than *must* as the modal verb. In addition, Rule 30 merely describes the need for ‘viable cultures’, while in Chapter 4, there is an advisory note that states that maintenance of type strains ‘may be by a variety of methods, e.g. in a medium, in a host by passage, in cells or exudates, or in the frozen or dried state’.

In the Approved Lists of Bacterial Names, there were over a dozen examples of species that can be propagated only in association with host cells, including *Chlamydia psittaci* and *Chlamydia trachomatis *, plus *Rickettsia typhi* and 11 other species of *Rickettsia* [4]. Since introduction of the requirement for deposition of type strains, dozens of additional names have been validly published for species of *Chlamydia* or *Rickettsia *, suggesting that most authorities take what Rule 18a describes as ‘pure culture’ to mean ‘viable culture’—an ambiguity that can be removed in subsequent editions of the Code.

However, such a move would exclude from *Candidatus* status any *Archaea* or *Bacteria* that can be stably propagated and deposited, but can be cultured only in association with another organism. Examples here include so-called *Candidatus* Nanosynbacter lyticus [30, 31], which can be cultured, but only in association with another bacterial species; *Nanoarchaeum equitans*, which can be cultured, but only in association with another archaeon [32]; and *Mycobacterium lepromatosis*, which can be cultured, but only in the footpads of nude mice [33, 34].

Similarly, there is no agreement on what counts as a satisfactory description of a *Candidatus* species. The examples provided in the initial proposal now appear dated, with their discussion of probes and primer sequences [10]. However, this has become less of an issue lately, as recent descriptions of *Candidatus* taxa have tended to converge on protologues similar in form to those used for cultured taxa, complete with etymological justifications for new names [35–38].

An initial weakness in the use of *Candidatus* names was the lack of any compilation of such names into published lists, despite a request for this in the original proposal and Appendix 11 of the Code [1, 10]. This meant that such names seldom underwent the linguistic quality control applied to validly published names of cultured taxa. However, after a first attempt at linguistic quality control for *Candidatus* names [39], this issue has now been addressed by annual publication of lists of *Candidatus* names within the IJSEM, together with suggested changes when names fail to comply with the rules and recommendations of the Code [20, 22].

However, it remains unclear how far suggested changes are actually taken up by authors, journals and databases. For example, when it was suggested that the genus name *Candidatus* Rohrkolberia should be changed to *Candidatus* Typhincola, the authors adopted an alternative name that they themselves crafted: *Candidatus* Symbiopectobacterium [40]. Similarly, the initially malformed *Borkfalki* was changed to *Candidatus* Borkfalkia, even though this clashed with Recommendation 6(10) that authors should not name taxa after themselves [36, 41]. However, similar issues also apply to the adoption of suggested changes to effectively published names, which may be ignored by authors if the original names merely flout recommendations, but do not break the rules of the Code. No more obvious example of a effectively published name that rides roughshod over the recommendations of the Code, but does not break the rules is *Myxococcus llanfairpwllgwyngyllgogerychwyrndrobwllllantysiliogogogochensis* [42].

A potential stylistic quibble is that adding the term *Candidatus* makes names longer than they have to be. However, for at least 20 years, *Candidatus* has been abbreviated in many publications to the simple two-letter moniker ‘*Ca*.’ [43]. Another criticism of the *Candidatus* option centres on its rather fussy and confusing orthographic requirements. Curiously, the Code does not mandate the use of italics for validly published Latin binomials or other taxonomic names, but in Appendix 11 does make clear: ‘the word *Candidatus*, but not the vernacular epithet is printed in italics’. However, a similar orthographic approach has been widely accepted in naming serovars of *Salmonella enterica *, where what was once considered a species epithet—but is now seen as merely a serovar—is presented in Roman type, for example, *Salmonella enterica* serovar Typhimurium or even *S*. Typhimurium [44].

Another source of uncertainty is whether a *Candidatus* name needs to be placed in quotation marks. Within the Code, some exemplar names are put quotation marks, whereas others are not. And among publications from 2021, some *Candidatus* names are published without quotation marks, e.g. *Candidatus* Sulfurimonas marisnigri [45], some sit within single quotation marks e.g. *‘Candidatus* Liberibacter asiaticus’ [46] and some sit within double quotation marks, e.g. *“Candidatus* Laterigemmans baculatus” [47]. Simplicity suggests that no such orthographic encumbrance is needed.

One final and perhaps most important weakness is that *Candidatus* names lack priority under the Code’s rules on valid publication. This can be seen as a fundamental flaw, when priority is deemed to be of central importance to systems of nomenclature, including not just the ICNP, but also in the equivalent codes for zoology and botany. As noted, so far, there have been no disputes over the priority of such names in the literature or in databases. But it remains unclear how problematic this will be when there are many thousands of *Candidatus* species names. Will authors, reviewers or editors play ‘nice’ and respect the general principle of scientific priority in this context or will there be a free-for-all with endless disputes? A recent case where names for cultured taxa published in peer-reviewed journals, but never validated, were subsequently overturned by a new set of authors [48] suggests this remains a potential problem for any name that is in use but not validly published—whether *Candidatus* or not.

However, this brings to mind a deeper philosophical question: should the status of names depend on cultivability? This can be seen to conflict with the opening principle of the ICNP: ‘Nothing in this Code may be construed to restrict the freedom of taxonomic thought or action’ and with General Consideration 5: ‘This Code of Nomenclature of Prokaryotes applies to *all* Prokaryotes’ (my italics). In addition, the requirement to use the term *Candidatus* has not been applied retrospectively, so it does not apply to the names of uncultured species published in the Approved Lists of Bacterial Names or to the thirty or more validly published names of uncultured organisms approved between 1980 and 2001 [4, 49–67]. These glaring inconsistencies sit uneasily within what is supposed to be a precise rule-governed system of nomenclature.

However, there is also a serious operational issue at stake. According to the strictest interpretation, assigning a *Candidatus* name to a taxon depends on proving a negative—being confident that the taxon has never been cultured by anyone anywhere in the world. To take a lively example that applies at the time of writing, let’s say we wished to assign a new *Candidatus* name to the genus given the designation CAG-485 by GTDB (https://gtdb.ecogenomic.org/searches?s=gt&q=CAG-485). Almost all of the more than 100 genomes classified within this genus represent metagenome-associated genomes, so it might appear safe to propose a new *Candidatus* name. However, only after exhaustively working through the metadata associated with all of the BioSamples associated with these genome sequences in the NCBI databases does it become clear that at least one of these (NCBI BioSample SAMN10878315) is in fact derived from a cultured isolate, so that some might argue the status *Candidatus* cannot be applied. A similarly exhausting process awaits anyone attempting to prove that well-established *Candidatus* taxa do not yet contain cultured relatives. In all such cases, it is probably best if we fall back upon the looser definition of *Candidatus*, ‘used for describing prokaryotic entities … for which characteristics required for description according to the Code are lacking’ and deny *Candidatus* status only when someone provides proof of culture in a peer-reviewed publication.

Scrutiny of genome sequences assigned to CAG-485 reveals an additional problem. Several of these originate from a study of the mouse gut microbiota conducted in Germany and are tagged as derived from cultured isolates [68]. However, on reading the paper it becomes clear that these represent genomes from ‘strains that could be isolated but failed being maintained in culture’. Should taxa based on such criteria be allowed *Candidatus* status? If so—as complying with the requirements for effective and valid publication of names for cultured taxa is far more time-consuming than publishing *Candidatus* names—those interesting in cataloguing microbial diversity may well favour approaches that avoid stable culture altogether and simply assign *Candidatus* (or even SeqCode) names to new found organisms.

## Opportunities

In the final chapter of the *Origin of Species*, Darwin prophesized: ‘Our classifications will come to be, as far as they can be so made, genealogies’ [69]. For plants and animals, this became a reality in the second half of the twentieth century with the adoption of cladistics, i.e. the scheme of phylogenetic taxonomy articulated by German entomologist Willi Hennig [70]. With the arrival of bacterial genome sequencing [71], terms and concepts from cladistics (e.g. clades and monophyletic groups) have permeated bacterial taxonomy [72], culminating in the near-universal adoption of sequence-based phylogenomic approaches [28]. Just as zoologists now accept that birds are dinosaurs, microbiologists now face similar challenges in say rejecting prokaryotes as a paraphyletic group [73–75] and accepting on cladistic grounds that mitochondria are bacteria and eukaryotes are archaea [76, 77].

This often unconscious appropriation of cladistics thinking has brought about a sea-change in microbial taxonomy—rather than an exhaustive description of phenotypic properties [78], the *sine qua non* in naming and describing a new species is now deposition of a genome sequence into the public domain [79], accompanied by a sequence-based circumscription built on a phylogenetic analysis of the type genome. In contrast to the chaos that predated the Approved List of Names, this means that it is now very easy to determine, through database searches, whether a bacterium characterized in say Europe does or does not belong to a taxon already defined in Asia or North America.

Such sequence-based comparisons provide a clear opportunity for ensuring that de facto priority of a name can be established by the deposition of a genome sequence into the public domain and a description of a taxon in a peer-reviewed publication. When twinned with Principle 1.1 of the Code ‘Aim at stability of names’, this effectively creates a default assumption that names of any taxa, including *Candidatus* taxa, should *not* be changed unless they conflict with any of the Code’s other Principles, Rules and Recommendations.

What this means is that although *Candidatus* names lack formal priority under the ICNP, there is in effect de facto establishment of priority through analyses of sequences. Thus, if I attempt to apply a new name to representatives of an existing *Candidatus* species, this will become obvious when I attempt classification using the NCBI or GTDB taxonomies and should, under the Code’s Principle 1.1, be blocked by reviewers, editors and the databases, unless I can provide a clear justification from the Code for overturning an existing name.

It is worth making a comparison here with names for bacterial and archaeal taxa published in peer-reviewed publications [80], but never *validly published*, which requires deposition of strains in two repositories and publication or listing in the IJSEM. Like *Candidatus* names, many of these have gained operational priority through sustained use—for example the name for the human pathogen *Tropheryma whipplei* [81], which has been used in over 600 publications listed in PubMed.

An alternative view is to see the absence of formal standing for *Candidatus* names not as a ‘bug’ but as a ‘feature’, as this means that they could be applied as provisional names to uncharacterized taxa, as an alternative to cumbersome alphanumeric labels, while leaving open the option that they could subsequently be changed after further characterization of taxa or changes in opinion.

The creation and curation of lists of *Candidatus* names published in the IJSEM provides a fresh opportunity for improving the linguistic quality of *Candidatus* names [20, 22]. From here, it is only a small step to encouraging authors, reviewers and editors to engage in linguistic quality control at the time such names are created, particularly as most errors are mundane problems (e.g. agreement in gender) that could be avoided through use of checklists—an approach that works even in high-risk contexts like medicine or aviation [82]—or through use of computer-based tools.

From my own experience, I can see that there is clearly scope for productive engagement with experts when creating *Candidatus* names *en masse*. Our recent progress with high-throughput name creation and quality control collaborating with an expert (Aharon Oren) provides proof-of-principle use of *Candidatus* names can be extended to cover huge numbers of new taxa discovered through metagenomic analyses. In an initial study of the chicken gut microbiome, we described names for 42 new *Candidatus* genera and 60 new *Candidatus* species [35]. Initially, this was done in a spreadsheet supplementary to the main manuscript. However, to ensure that names were propagated in lists and databases, conventional protologues were subsequently published in a corrigendum [83].

In a subsequent study, we described 657 *Candidatus* species names and 158 *Candidatus* genera names in a series of protologues that occupied over 100 pages of the paper [37]. These efforts primed a more ambitious study, which included software for generation of names and protologues, together with creation of over a million well-formed names for bacterial species [38]. Such efforts show that generating names for large numbers of taxa—whether cultured or uncultured—is indeed scalable in the age of the big-data metagenomics. The fact that a new version of the Code is now in preparation [84] also brings another opportunity to revisit and refine the *Candidatus* approach while clarifying ambiguities or contradictions in the current version of the Code (as detailed above). There would be an opportunity to solve the problem of priority, if the *Candidatus* status were brought within the main body of the Code, with Rules on its application, name formation, designation of type material etc., plus a Rule that if a *Candidatus* taxon is brought into culture then the name for the type species must be conserved.

## Threats

A key challenge to continued use of the status *Candidatus* comes from the planned launch of a new code of nomenclature for bacteria and archaea termed the SeqCode [17, 19], which proposes to remove the need for deposition of type strains in culture collections, but instead to give *de jure* priority to names associated with a type sequence.

In preferring revolution to evolution, such an approach ignores the current *status quo*, in which adherence to the Code’s requirement for stability twinned with ease of database searches means that *Candidatus* names already have de facto priority in the literature. The success of this new venture will depend on whether authors, journals, editors and online resources (e.g. LPSN and NCBI taxonomy) will pay more attention to names issued under the new code than they already grant to *Candidatus* names. Similarly, uncertainties remain over whether the new code aims to govern nomenclature of all taxa of *Bacteria* and *Archaea* or only the uncultured. Clearly if the SeqCode is going to cover only uncultured organisms, this will require exhaustive checks to see which code should apply to which organisms and will require clarity on how conflicts with the ICNP will be managed.

Will the two codes run in parallel, with some authorities, e.g. NCBI, LPSN or the authors of a recent opinion piece [85], sticking with the *Candidatus* option, while others abandon it, just as GTDB ignores it already? Under this scenario, at least some microbiologists will continue to use the *Candidatus* option for years to come. More generally, only time will tell whether the SeqCode will see widespread acceptance or fall by the wayside, as has happened with similar efforts such as the PhyloCode and the BioCode [86, 87].

A potential operational challenge to the status quo comes from increasingly unsustainability in practice of the current approach to nomenclature. The requirement for deposition in culture collections in two countries has become more difficult under national and international rules on intellectual property, including the Nagoya protocol [88]—although if digital sequence information is seen as a genetic resource, the Nagoya protocol might also create problems for releasing genome sequences as type material.

Recent publications, in which names for novel species and genera have been published without deposition in two repositories, show that even those employed in culture collections do, on occasion, ignore the ICNP’s rules for effective publication [68, 89]. Furthermore, breakthroughs in culturing previously uncultured organisms, including co-culture and dilution-to-extinction culture [90] are probably not yet compatible with strain deposition as currently configured. All this suggests a re-think of the current rules may become necessary over the coming years, so that a subsequent vote on whether sequences can become type material may turn out differently from the most recent episode and sweep away the need for the *Candidatus* status.

## Conclusions

The *Candidatus* option clearly works, as evidenced by a quarter century of use. What’s more, it has been shown to cope with modern high-throughput approaches to taxonomic discovery. Although lacking priority under the code, many *Candidatus* names have already achieved standing in the academic literature and in key databases and there is a clear path to valid publication of such names on culture. Continued use of Candidatus names provides an alternative to the upheaval that might accompany creation of a new additional code of nomenclature. As we have a solution that already works for naming uncultured organisms, faced with the calls for change, the pragmatist will probably want to invoke the Code’s opening call ‘Aim for stability’ and say if ‘it ain't broke, why fix it?’.

It is worth stressing that *Candidatus* names are not currently in competition with other well-formed names, but instead with an unpalatable alphanumerical spaghetti, epitomized in GTDB designations such as UBA6965 or sp000063525. We now face the urgent and exhilarating challenge of creating the many thousands, if not millions, of new well-formed Latin names for newly discovered species. So my own view is let’s get on with creating the names and let posterity decide whether they need to be prefaced with *Candidatus. Carpe diem!*
